# Immune modulation in inflammatory bowel disease: therapeutic promise of baicalein

**DOI:** 10.3389/fphar.2025.1723606

**Published:** 2025-12-10

**Authors:** Yang Wang, Luyao Zeng

**Affiliations:** 1 The Seventh People’s Hospital of Chongqing/The Central Hospital Affiliated to Chongqing University of Technology, Chongqing, China; 2 Gastrointestinal Surgery, The Second Affiliated Hospital of Chongqing Medical University, Chongqing, China

**Keywords:** baicalein, inflammatory bowel disease, intestinal barrier, immunomodulation, gut microbiota

## Abstract

Inflammatory bowel disease (IBD) remains a major global health burden, driven by a multifaceted pathogenesis that includes immune dysregulation, epithelial barrier disruption, oxidative stress, and gut microbiota imbalance. Addressing these interconnected processes requires multi-targeted therapeutic strategies that go beyond conventional single-pathway interventions. Baicalein, a key flavonoid derived from *Scutellaria baicalensis* (Huang Qin), has emerged as a promising candidate due to its broad-spectrum pharmacological properties. This review synthesizes current advances in understanding how baicalein exerts therapeutic effects against IBD through an integrated network of mechanisms. These include potent suppression of inflammatory signaling and oxidative stress, restoration of epithelial integrity via modulation of tight junction proteins and the MLCK/p-MLC2 pathway, and reprogramming of dysregulated immune circuits by rebalancing T-cell subsets and macrophage polarization. In addition, baicalein mitigates pathological cell death pathways such as ferroptosis and pyroptosis and orchestrates beneficial shifts in the gut microbiota–metabolite axis. By bridging classical anti-inflammatory mechanisms with emerging immunoregulatory and microbiome-targeted insights, this review highlights baicalein as a potential multi-dimensional therapeutic strategy for IBD and outlines future directions for its clinical translation.

## 1 Introduction: the therapeutic challenge of IBD and the promise of baicalein

Inflammatory Bowel Disease (IBD), encompassing Crohn’s disease and ulcerative colitis, is a chronic, debilitating condition affecting millions worldwide, with a notably rising incidence (Gilliland et al., 2024; Gracie et al., 2019). The disease etiology is multifactorial, rooted in a complex interplay of genetic susceptibility, environmental factors, a dysregulated immune response, and a disrupted gut microbiome (Barberio et al., 2021; Bisgaard et al., 2022). A particularly alarming aspect of IBD is its high rate of psychiatric comorbidities, such as anxiety and depression, which are 2–3 times more prevalent in IBD patients than in the general population (Chen et al., 2020; Cryan et al., 2019). This underscores the systemic nature of the disease and the involvement of the gut-brain axis, potentially driven by microbiota dysbiosis, metabolic imbalances, and increased gut barrier permeability (Agirman and SnapShot, 2021; Craig et al., 2022; Sharon et al., 2016).

The limitations of conventional therapies—including 5-aminosalicylates, corticosteroids, and biologics—highlight the urgent need for novel, multi-targeted treatment strategies. In this context, natural products derived from traditional medicine offer a rich source of potential therapeutics (Zhao et al., 2019). The root of *Scutellaria baicalensis* Georgi, known as Huang Qin in Chinese medicine, has a documented history of use spanning over 2,000 years, primarily for treating “damp-heat” dysentery and other gastrointestinal ailments. Its reputation as an “intestinal-targeting” herb is supported by modern pharmacological evidence (Shen et al., 2020).

The therapeutic efficacy of *S. baicalensis* is largely attributed to its rich content of flavonoids, with baicalin and its aglycone counterpart, baicalein, being the most prominent and well-studied (Hu et al., 2021). These compounds are known for their wide range of biological activities, including anti-inflammatory, antioxidant, antimicrobial, and immunomodulatory effects (Xu et al., 2020). This review aims to synthesize recent advances in understanding the pharmacological mechanisms of baicalein (and its precursor baicalin, which is often metabolized to baicalein in the gut) in the context of IBD treatment, providing a comprehensive and updated reference for future investigations (Li J. et al., 2024).

## 2 Fundamental pharmacological mechanisms of baicalein in IBD

### 2.1 Immune heterogeneity in IBD

A central challenge in IBD is not simply “too much inflammation,” but a mosaic of immune cell states—across epithelium, stroma, and multiple immune lineages—that vary between patients and even between locations in the same colon. Single-cell atlases of ulcerative colitis (UC) mucosa show dozens of epithelial, stromal, and immune subsets with disease-specific reprogramming. For example, Smillie et al. profiled colonic cells and described disease-associated inflammatory fibroblasts (IL13RA2^+^IL11^+^), altered epithelial subsets (e.g., BEST4^+^ enterocytes), and immune–stromal circuits linked to anti-TNF resistance. These findings argue that therapeutic response hinges on which cell states dominate in a given patient rather than a single pathway “on/off” model (Smillie et al., 2019).

Beyond static snapshots, longitudinal single-cell studies across anti-TNF therapy show that mucosal compartments are dynamic ecosystems: inflammatory myeloid states, pathogenic Th17 programs, and epithelial stress modules wax and wane with treatment. Some patients fail to extinguish IL-23–responsive T-cell programs despite anti-TNF, pointing to cytokine-network plasticity that sustains disease activity (Thomas et al., 2024; Atreya and Neurath, 2022).

Mechanistically, IL-23–responsive, apoptosis-resistant TNFR2^+^IL-23R^+^ T cells are enriched in anti-TNF non-responders, highlighting a distinct, drug-refractory T-cell niche that may require IL-23–pathway blockade. Newer clinical directions (e.g., risankizumab) build directly on this axis (Schmitt et al., 2019; Bourgonje et al., 2025).

Another layer of heterogeneity involves tissue-resident memory T cells (T_RM). These cells persist in mucosa after flares and are implicated in relapse biology, with region-specific T_RM states along intestinal axes and evidence that imbalanced CD8^+^/CD4^+^ T_RM pools contribute to chronic, relapsing inflammation. Recent spatial/single-cell work further shows epithelial metaplasia can recruit T cells and neutrophils, adding an epithelial–immune loop to heterogeneity (Lutter et al., 2021; Xia et al., 2024; Oliver et al., 2024).

Although single-cell trials of baicalein in patients are lacking, its multi-node actions map onto the heterogeneous niches above:

1. Th17/Treg axis and IL-23 circuitry. Preclinical data show baicalin/baicalein reduce Th17 and IFN-γ/IL-17 outputs while promoting Treg/IL-10, aligning with the need to curb IL-23-responsive effector programs that underlie anti-TNF resistance (Atreya et al., 2022; Schmitt et al., 2019).
2. PMC Myeloid polarization. Baicalin can reprogram macrophages toward M2 and dampen TLR4–IRF/STAT signaling, shrinking inflammatory monocyte-derived niches that fuel epithelial injury (Liang et al., 2019).
3. Microbiota–metabolite control of immune states. By enriching *Lactobacillus* and other beneficial taxa and increasing SCFAs/indoles, baicalin/baicalein engage AhR/IL-22 and epigenetic programs known to shape DC tolerogenesis and T-cell plasticity—mechanisms that plausibly re-tilt the mosaic toward homeostasis (Zhang S. et al., 2025).

These lines of evidence suggest baicalein is not just an “anti-inflammatory,” but a niche-level modulator capable of reshaping immune composition and state, especially when combined with pathway-targeted biologics (e.g., IL-23 blockade) to address patient-specific dominant circuits. Integrating single-cell readouts (myeloid states, T_RM signatures, IL-23–responsive T cells) into preclinical and early clinical studies of baicalein would directly test this “heterogeneity-aware” hypothesis and help identify responders.

### 2.2 Suppression of inflammatory signaling and oxidative stress

The anti-inflammatory activity of baicalein constitutes one of the cornerstones of its therapeutic effect (Kim et al., 2020; Lai et al., 2024; Li W. et al., 2022; Li Y. Y. et al., 2024). *In vitro* and *in vivo* studies consistently demonstrate its capacity to dampen the inflammatory cascade. Baicalein effectively inhibits the activation of key transcription factors such as Nuclear Factor-kappa B (NF-κB) and mitogen-activated protein kinase (MAPK) pathways, leading to a significant reduction in the production of pro-inflammatory cytokines, including Tumor Necrosis Factor-alpha (TNF-α), Interleukin-1 beta (IL-1β), and Interleukin-6 (IL-6) (Cui et al., 2014; Ciesielska et al., 2021; Hu et al., 2020; Loi et al., 2023). In murine models of colitis induced by dextran sulfate sodium (DSS), baicalein treatment markedly lowers colonic levels of these cytokines, reduces neutrophil infiltration (as evidenced by decreased myeloperoxidase (MPO) activity), and improves histological damage scores (Zhang et al., 2017; Feng et al., 2014; Dou et al., 2012).

Beyond cytokine modulation, baicalein targets enzymes central to the inflammatory process. It suppresses the expression of inducible nitric oxide synthase (iNOS) and cyclooxygenase-2 (COX-2), thereby reducing the production of nitric oxide (NO) and prostaglandin E2 (PGE2) (Zhong et al., 2019; Luo et al., 2017). Notably, its anti-inflammatory action also involves the balanced inhibition of both COX and 5-lipoxygenase (5-LOX) pathways, regulating the synthesis of a broader spectrum of eicosanoids like leukotriene B4 (LTB-4) and thromboxane B2 (TXB-2) (Pallio et al., 2016; Neurath, 2014).

Concurrently, baicalein counteracts the pervasive oxidative stress in IBD. It functions as a direct reactive oxygen species (ROS) scavenger, neutralizing superoxide anions and hydroxyl radicals, an activity attributed to its phenolic hydroxyl groups (Formiga et al., 2020). Moreover, it bolsters the endogenous antioxidant defense system by enhancing the activity of key enzymes such as superoxide dismutase (SOD), catalase (CAT), and glutathione peroxidase (GPx) (Yao et al., 2016; Pedersen et al., 2014). This dual anti-inflammatory and antioxidant action disrupts the vicious cycle where inflammation begets oxidative stress and *vice versa*, thereby mitigating extensive tissue damage.

### 2.3 Reinforcement of the intestinal epithelial barrier

A compromised intestinal barrier is a hallmark of IBD pathology. Baicalein addresses this critical aspect through multiple synergistic mechanisms. Firstly, it exerts a direct cytoprotective effect on intestinal epithelial cells (e.g., Caco-2 cells), shielding them from apoptosis induced by inflammatory mediators like TNF-α and from oxidative insult, thereby preserving epithelial integrity (Ma et al., 2020; Seyed et al., 2019; Song et al., 2021).

Secondly, and most importantly, baicalein positively regulates the expression and distribution of tight junction (TJ) proteins, which are the fundamental structural components of the paracellular barrier (Stockwell et al., 2017). It upregulates the levels of occludin, claudins, and zonula occludens-1 (ZO-1) (Wang et al., 2017). This action is closely linked to its inhibition of the myosin light chain kinase (MLCK)/phosphorylated myosin light chain 2 (p-MLC2) pathway (Huang et al., 2020). Activation of this pathway leads to cytoskeletal contraction and internalization of TJ proteins, increasing permeability. By inhibiting MLCK phosphorylation, baicalein helps stabilize the perijunctional actin-myosin ring, preventing the breakdown of TJs (Dokladny et al., 2016; Vučković et al., 2021). This mechanistic insight is corroborated by studies showing that wogonoside, a closely related flavonoid from Scutellaria, protects barrier function via the same pathway. The functional improvement in barrier integrity is further confirmed by measurements showing reduced serum levels of D-lactate and endotoxin following baicalein treatment in experimental colitis models (Leung et al., 2016; Xiao-lan et al., 2020).

## 3 Emerging frontiers: novel mechanisms of action

### 3.1 Inhibition of non-apoptotic cell death pathways

Recent research has unveiled the significant roles of ferroptosis and pyroptosis in IBD pathogenesis, and baicalein shows promise in countering these processes.

- Ferroptosis: This is an iron-dependent form of regulated cell death driven by lipid peroxidation. In DSS-induced colitis models, baicalein treatment upregulates key anti-ferroptotic defense molecules, including glutathione peroxidase 4 (GPX4), the cystine/glutamate antiporter subunit SLC7A11, and the transcription factor Nrf2. Concurrently, it suppresses the expression of acyl-CoA synthetase long-chain family member 4 (ACSL4), a pro-ferroptotic enzyme. By bolstering cellular defenses against lipid peroxidation, baicalein protects intestinal epithelial cells from ferroptotic death, preserving mucosal integrity (Lv et al., 2025; Zhang J. et al., 2025; Ocansey et al., 2023).
- Pyroptosis: This is a highly inflammatory form of programmed cell death mediated by gasdermin D (GSDMD). Studies on baicalein derivatives indicate that they can inhibit the NLRP3 inflammasome/caspase-1/GSDMD signaling axis. By doing so, they reduce the cleavage of GSDMD and the subsequent maturation and release of pro-inflammatory cytokines IL-1β and IL-18, thereby mitigating inflammation and associated tissue damage (Ocansey et al., 2023; Guo et al., 2025; Wang et al., 2021; Yao et al., 2022).

### 3.2 Remodeling the gut microbiome and metabolite axis

Gut dysbiosis is a central driver of IBD. Baicalein administration has been demonstrated to induce a beneficial shift in the gut microbial community structure. It consistently promotes the enrichment of beneficial bacterial taxa, such as *Ligilactobacillus* and Lachnospiraceae *NK4A136 group*, while suppressing the abundance of potential pathobionts like *Escherichia-Shigella* (Rahman et al., 2021).

This ecological restructuring has profound functional consequences. The restored beneficial microbiota increases the production of key microbial metabolites, particularly short-chain fatty acids (SCFAs, e.g., butyrate) and indoles (Wang et al., 2019; Wu et al., 2019; Zhu et al., 2020). These metabolites are not merely waste products; they function as crucial signaling molecules. For instance, indoles activate the aryl hydrocarbon receptor (AhR) on intestinal epithelial cells and group 3 innate lymphoid cells (ILC3s) (Zhu et al., 2018; Liu et al., 2020; Li Y. Y. et al., 2022). This activation stimulates the production of interleukin-22 (IL-22), a cytokine pivotal for promoting epithelial cell proliferation, regeneration, and barrier repair. Thus, baicalein establishes a positive, self-reinforcing feedback loop—the “microbiota-metabolite-barrier” axis—whereby its direct actions on the host are amplified through beneficial modulation of the gut ecosystem, contributing to long-term mucosal stability (Ye et al., 2022; Hung et al., 2018; Arnott et al., 2000; Ayoub et al., 2024). A summary of the principal mechanisms and molecular targets of baicalein in IBD is provided in Table 1.

**TABLE 1:** Summary of the principal mechanisms and molecular targets of baicalein in IBD.

Mechanistic category	Key molecular targets /pathways	Representative findings
Suppression of inflammatory signaling	NF-κB, MAPK, TLR4, IL-1β, IL-6, TNF-α	Reduces cytokine release, downregulates TLR4/NF-κB axis, inhibits MAPK phosphorylation (Kim et al., 2020; Lai et al., 2024; Li et al., 2022a; Li et al., 2024b; Cui et al., 2014; Ciesielska et al., 2021; Hu et al., 2020; Loi et al., 2023; Zhang et al., 2017; Feng et al., 2014; Dou et al., 2012)
Regulation of Th17/Treg axis	IL-23/STAT3, RORγt Foxp3	Shifts Th17→Treg balance reduces IL-17/IFN-γ levels, increases IL-10 (Atreya et al., 2022; Schmitt et al., 2019; Zhu et al., 2018; Liu et al., 2020; Li et al., 2022b)
Anti-ferroptosis	GPX4 ↑, SLC7A11 ↑ ACSL4 ↓, Nrf2 ↑	Prevents lipid peroxidation and protects epithelial cells (Lv et al., 2025; Zhang et al., 2025b; Ocansey et al., 2023)
Anti-pyroptosis	NLRP3, Caspase-1, Gasdermin D	Reduces IL-1β/IL-18 production and mitigates inflammatory cell death (Ocansey et al., 2023; Guo et al., 2025; Wang et al., 2021; Yao et al., 2022)
Barrier protection (TJ proteins)	Occludin, Claudins, ZO-1, MLCK/p-MLC2	Prevents TJ disruption, improves paracellular integrity (Stockwell et al., 2017; Wang et al., 2017; Huang et al., 2020; Dokladny et al., 2016; Vučković et al., 2021)
Oxidative stress suppression	SOD, CAT, GPx, ROS neutralization	Restores antioxidant system and reduces epithelial injury (Formiga et al., 2020; Yao et al., 2016; Pedersen et al., 2014)
Microbiota–metabolite modulation	SCFAs (butyrate) indoles, AhR-IL-22	Enhances beneficial taxa; increases IL-22 and epithelial repair (Zhu et al., 2018; Liu et al., 2020; Li et al., 2022b)

## 4 Translational considerations and future perspectives

The translation of baicalein’s promising preclinical results into clinical practice requires careful consideration of several factors. Systematic analysis of animal studies suggests an effective dose range for baicalin/baicalein is approximately 60–150 mg/kg, with treatment durations typically around 10–14 days yielding consistent improvements across multiple biomarkers (e.g., MPO, NF-κB, caspase-3). This provides a foundational PK/PD framework for future human trials (Chen et al., 2012; Yang et al., 2013; Burisch et al., 2013; Sun et al., 2015).

A particularly promising avenue lies in combination therapy. Exploring the synergistic effects of baicalein with standard first-line drugs like 5-aminosalicylates (5-ASA) could lead to regimens that enhance efficacy, allow for dose reduction of conventional drugs (minimizing side effects), and potentially overcome therapeutic resistance (Magro et al., 2020).

Furthermore, addressing the challenges of bioavailability and targeted delivery is paramount. The development of advanced drug delivery systems, such as colon-targeted formulations (e.g., pH-dependent capsules, microbiota-triggered nanoparticles), could ensure that an optimal concentration of the active compound (be it baicalin or the more potent baicalein) is delivered precisely to the site of inflammation in the colon, thereby maximizing therapeutic efficacy.

## 5 Conclusion

Baicalein represents a paradigm for a multi-target, holistic approach to IBD management. Its therapeutic efficacy stems not from a single, isolated action, but from a synergistic network of interconnected mechanisms: quenching inflammation and oxidative stress, fortifying the intestinal physical barrier, recalibrating the dysregulated immune landscape, intercepting novel cell death pathways, and beneficially remodeling the gut microbiome. This comprehensive pharmacological profile allows it to address the complex, multifactorial nature of IBD more effectively than single-target agents. Future research should prioritize well-designed clinical trials to validate its efficacy and safety in humans, alongside continued efforts in pharmaceutical engineering to optimize its delivery. Baicalein, deeply rooted in traditional medicine and validated by modern science, holds significant promise as a valuable component in the future arsenal against IBD.
